# Preparation and characterization of lemongrass oil nanoemulsion: Antimicrobial, antibiofilm, antioxidant, and anticancer activities

**DOI:** 10.1515/biol-2025-1159

**Published:** 2025-08-28

**Authors:** Mohamed T. Selim, Bahaa M. Badr, Salem S. Salem, Fathy M. Elkady, Mostafa A. Abdel-Maksoud, Nasser Ibrahim Issa, Karim M. Sobhy, Khaled M. Shaban, Ahmed A. Abdallah, Ali M. Sabeq, Abdulaziz Alamri, Mohamed Y. Zaky, Abeer S. Aloufi, Amr H. Hashem

**Affiliations:** Botany and Microbiology Department, Faculty of Science, Al-Azhar University, Nasr City, Cairo, 11884, Egypt; Department of Basic Medical and Dental Sciences, Faculty of Dentistry, Zarqa University, Zarqa, Jordan; Department of Medical Microbiology and Immunology, Faculty of Medicine, Al-Azhar University (Assiut branch), P.O. Box 71524, Assiut, Egypt; Microbiology and Immunology Department, Faculty of Pharmacy (Boys), Al-Azhar University, P.O. Box 11884, Cairo, Egypt; Department of Botany and Microbiology, College of Science, King Saud University, P.O. 2455, Riyadh, 11451, Saudi Arabia; Biochemistry Department- College of Science- King Saud University, P.O. Box 2455, Riyadh, 11451, Saudi Arabia; UPMC Hillman Cancer Center, Division of Hematology and Oncology, Department of Medicine, University of Pittsburgh, Pittsburgh, PA, 15213, United States of America; Department of Biology, College of Science, Princess Nourah bint Abdulrahman University, P.O. Box 84428, Riyadh, 11671, Saudi Arabia

**Keywords:** lemongrass essential oil, nanoemulsions, antioxidant, antimicrobial, antibiofilm, cytotoxicity

## Abstract

Although citrus essential oils, including lemongrass essential oil, have antibacterial, anti-biofilm, and antioxidant properties, their biological instability and poor water solubility render them unsuitable for industrial usage. Thus, this study aimed to prepare both lemongrass essential oil emulsion (LEO-E) and lemongrass essential oil nanoemulsion (LEO-NE), and evaluate their different bioactivities. Characterization by gas chromatography–mass spectroscopy (GC–MS) and evaluation of antimicrobial, antibiofilm, antioxidant, and anticancer activities were carried out. GC–MS results illustrated that D-limonene compound is the dominant among other compounds in LEO. According to transmission electron microscopy and dynamic light scattering, LEO-NE appeared as spherical-shaped droplets with a constant size spanning from 29.1 to 37.4 nm with a polydispersity index value of 0.163. Antimicrobial results showed that LEO-NE exhibited promising antimicrobial activity against *Bacillus subtilis, Staphylococcus aureus, Escherichia coli, Pseudomonas aeruginosa, Candida albicans*, and *Austromerope brasiliensis* with inhibition zones of 25.33 ± 1.1, 26.5 ± 0.5, 22 ± 1, 24.33 ± 0.5, 28.6 ± 1, and 27.97 ± 0.9 mm, respectively. Moreover, LEO-NE showed considerable antiـbiofilm efficacy toward *S. aureus* and *P. aeruginosa* with inhibition percentages at 1/2 of MIC of 98.92 and 92.62%, respectively. Furthermore, LEO-NE exhibited antioxidant activity using the 2,2-diphenyl-1-picrylhydrazyl method with 88.5% at 100 µg/mL concentration. In addition, LEO-NE displayed potential anticancer activity toward the human prostate cancer cell line (PC3) and human liver cancer cell line (Hep-G2), where IC_50_ values were 170.09 and 105.06%, respectively. In conclusion, the prepared LEO-NE in the current study had antimicrobial, antibiofilm, antioxidant, and anticancer activities, which can be used in the medical and pharmaceutical fields.

## Graphical abstract

**Figure:**
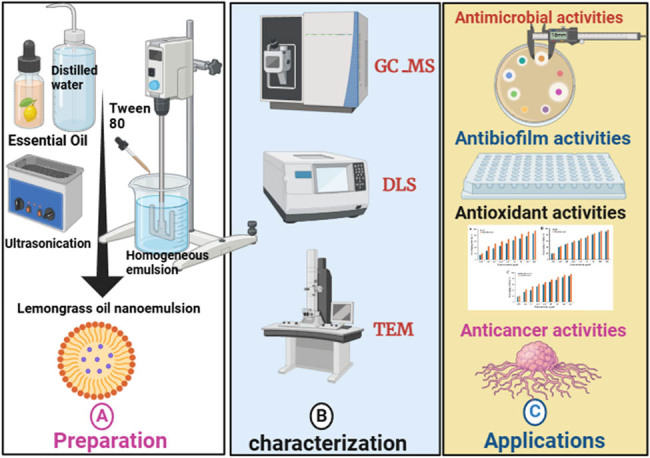

## 1 Introduction

The current world is fortunate with rapid sophisticated technologies for administering medications for the treatment of various disorders [1]. These developments have improved our level of living and promoted healthy lifestyles. However, as people become more aware of the negative consequences of chemically based pharmaceuticals on human health, they are increasingly seeking natural alternatives to cure a variety of disorders [2]. Reports indicate that more than 80% of people on the planet today take their medications from plants or herbs. More than 9,000 varieties of plants have been identified for their medicinal uses, and over 1,500 species have been identified for their flavorful and fragrant qualities [3].

Lemongrass essential oil (LEO) is mostly produced from lemongrass using cold pressing or steam distillation. It contains about 200 chemicals, with more than 85% of them being volatile. The majority of LEO is made up of oxygenated compounds with antiviral, anticancer, antioxidant, bactericidal, and inflammatory qualities, such as esters, alcohols, ketones, terpenoids, and aldehydes [4,5]. To prevent oxidation and the bacterial rotting impact, LEO was developed as a green safeguard and used in the preservation of meat and produce [6]. LEO’s effects on kiwifruit soft-rotting fungi have not been well documented, despite a recent study showing that it has useful inhibitory influence on foodborne fungi [7].

The LEO’s water insolubility, among other drawbacks including environmental sensitivity, low stability, and volatility is the biggest obstacle to its widespread application. To lessen its hydrophobicity, emulsions could include LEO; however, conventional emulsions are thermodynamically unstable, and their constituents have a tendency to separate [8]. Emerging nanotechnology can be used to make nanoemulsions (NEs), which can solve these problems [9]. One kind of drug delivery system is an NE, which has a consistent formulation quality, an easy production technique, and possesses some thermodynamic and kinetic stability, which can lessen drug delivery irritation and, on the other, successfully increase the drug stability following emulsification [10]. The NEs have droplets that range from 20 to 200 nm [11]. In the meantime, the surface and interface characteristics of NEs are determined by their particle size. Small particle NEs have a high surface-to-volume ratio, low particle weight, and the ability to defy gravity through Brownian motion, which can lessen the likelihood of flocculation, aggregation, and coalescence [12]. Therefore, this research’s main goal was to prepare both lemongrass essential oil emulsion (LEO-E) and lemongrass essential oil nanoemulsion (LEO-NE), and evaluation of their anti_microbial, antibiofilm, anti_oxidant, and anticancer activities.

## 2 Materials and methods

### 2.1 Preparation of LEO-NE

The LEOs were purchased from Sigma Aldrich (Sigma, USA). A mixture of 10 mL essential oil and 250 mL distilled water was centrifuged at room temperature for 10 min at 5,000 rpm using a high-speed homogenizer (F6/10, Jingxin, China). During this phase, 20 mL of Tween 80 was gently added to create a homogeneous emulsion [2]. The resulting crude emulsion was sonicated with an ultrasonicator (JY92-11D, Jingxin, China). The sonication time was standardized at 25 min in all circumstances. Ultrasonication provides strong and disruptive forces that produce the NE droplets [13].

### 2.2 Characterization of LEO-NE

#### 2.2.1 Gas chromatography–mass spectroscopy (GC–MS)

GC–MS analysis was applied to identify the functional constituents in LEO and their makeup, in accordance with previously published protocols [14,15]. In the capillary column (30 m × 0.25 mm × 0.25 μm), LEO was injected. Helium gas, with a constant flow rate of 1 mL min^−1^, was employed as a carrier gas. Following a 1 min hold at 40°C, the temperature was programmed to increase by 3°C per minute to 220°C over the following 25 min, culminating in a 10 min hold at 250°C. The ion source temperature was set to 230°C, while MS conditions were established at 70 eV EI. An atomic unit range of 35–350 was chosen for the mass charge. By utilizing the retention durations and comparing the value (mass spectra) of the eluted molecules with a collection of recognized standard chemicals’ spectra, the compounds were identified.

#### 2.2.2 Measurements of LEO-NE particle size

Using dynamic light scattering (DLS), the average particle size and polydispersity index (PDI) of the nanodroplets were measured. The intensity dispersal value was used to compute the *z*-average size. To reduce scattering effects, samples were diluted with purified water before measurements. All measurements were established three times to obtain an average value.

Additionally, LEO-NEs were diluted with purified water to avoid inter-particle aggregation and subsequently filtered using a filter paper. Samples were then absorbed onto copper grids covered with carbon for a minute at room temperature. Afterward, the samples were stained negatively so that a transmission electron microscope could examine the morphology of the generated LEO-NE.

### 2.3 Antimicrobial activity

To explore the antimicrobial properties of LEO-NEs, various pathogenic strains were tested. These included Gram-positive bacteria (*Staphylococcus aureus* – ATCC 6538 and *Bacillus subtilis* – ATCC-6633), Gram-negative bacteria (*Pseudomonas aeruginosa* – ATCC 9027 and *Escherichia coli* – ATCC 11229), and eukaryotic strains such as unicellular fungi (*Candida albicans* – ATCC 10231) and multiـcellular fungi (*Austromerope brasiliensis* – ATCC 16404). The agar well-diffusion method was carried out to assess the antimicrobial activity [16]. Initially, the growth of specific bacteria, yeast cell, and *A. brasiliensis* strains was recultured using nutrient agar and potato dextrose agar plates, respectively. The plates were incubated for 24 h at 35 ± 2°C for bacterial cells and yeast, and for 72 h at 28°C for *A. brasiliensis*. The freshly grown bacterial and yeast strains were then transferred to plates containing Mueller–Hinton agar for bacterial cells and yeast and to plates containing Czapek Dox agar for the *A. brasiliensis* standard strain. Three wells, each 0.6 mm in diameter, were made on each agar plate, utilizing a sterile cork borer. Then, a 1:1 mixture of 100 µL of each of LEO-E and LEO-NEs were added to the wells. After an hour of refrigeration, the plates containing cells with LEO-NEs were incubated at 35 ± 2°C for 24 h. Each well’s surrounding inhibitory zone’s diameter was taken in millimeters (mm). In triplicate, the task at hand was conducted.

### 2.4 Antioxidant activity of LEO-NE

The 2,2-diphenyl-1-picrylhydrazyl (DPPH) assay was employed to evaluate and distinguish between the free-radical-scavenging ability of LEO-NEs and LEO-E. For the analysis, a test tube was prepared with 1 mL of DPPH, 450 µL of Tris–HCl buffer (pH 7.4), and 1 mL of the tested LEO-NEs or LEO-E solution. Once the mixture was thoroughly combined, it was incubated at 37°C for 30 min with vigorous shaking at 100 rpm in the dark. Another set of experiments was performed under the same conditions and concentrations, using a positive control (ascorbic acid). For the negative control, DPPH with Tris buffer in a specimen tube was used in the same setup but without any treatment (LEO-NEs, LEO-E, or ascorbic acid). Following the incubation stage, the rate of absorption of the generated color at 517 nm was assessed. The following formulas were used to get the percentages of free radical scavenging [17].

\[\begin{array}{c}\text{DPPH}\hspace{.25em}\text{scavenging}\hspace{.25em}\text{\}\%\hspace{.25em}=\text{absorbance}\hspace{.25em}\text{values}\hspace{.25em}\text{of}\hspace{.25em}\text{control}\\ \hspace{1em}-\text{absorbance}\hspace{.25em}\text{values}\hspace{.25em}\text{of}\hspace{.25em}\text{treatment/absorbance}\\ \hspace{1em}\text{values}\hspace{.25em}\text{of}\hspace{.25em}\text{control}\times \text{100}\text{.}\end{array}]\]

### 2.5 Antibiofilm effect of LEO-NE

Bacterial isolates were inoculated in tryptic soy broth (TSB) containing 1% w/v glucose and cultured for 24 h at 37°C to evaluate the inhibitory action of the tested compounds on bacterial biofilms. Following that, the bacterial suspensions of cells were diluted to a turbidity of 0.5 McFarland standards. Before the bacterial inoculum was added, 200 µL of TSB containing glucose (1% w/v) was applied to 96-well flat-bottom microtiter plates. Sub-inhibitory gradient dosages of LEO-NEs, LEO-E, or their mixture were applied to the plates, and they were subsequently incubated for 24 h at 37°C. Following incubation, the contents of the wells were removed, and 200 µL of crystal violet (CV; 0.1% w/v) was stained for 20 min at 37°C. The wells were then fixed for 10 min in methanol (99%) and twice cleaned in phosphate buffered saline (pH 7.4). The plate was cleansed with sterile distilled water, and it was permitted to dry at room temperature after the extra CV was removed. To quantify the suppression of biofilm formation, the dye was dissolved in 200 µL of 30% acetic acid and subsequently added to each well. A microplate reader was employed to measure the optical density (OD) at 492 nm. At least three replicates of each test sample were conducted [18–20]. The percentage (%) of inhibition was monitored utilizing the following formula:

\[{\mathrm{Inhibition}} \% =\frac{{\mathrm{OD\; growth}}-{\mathrm{OD\; sample}}}{{\mathrm{OD\; growth\; control}}}{\mathrm{\times }}100.]\]

### 2.6 Cytotoxic effect of LEO-NE

Investigation of the potential cytotoxicity effect of LEO and LEO-NEs was carried out on the WI-38 normal cell line, and its antitumor effect was studied against PC3 and Hep-G2 cancerous cell lines by the MTT reduction assay. In this assay, cells treated with different concentrations of LEO-NEs (500, 250, 125, 62.5, or 31.25 µg/mL) were checked in triplicate culture for each concentration. A colorimetric assay was used to measure the cell viability. A two-fold diluted sample was made in RPMI medium + 2% serum, and then 0.1 mL from every dilution was tested in different wells. Wells with cell suspension without the tested compounds were considered as a negative control. The MTT solution (20 µL) was added to each well. The plates were incubated with 5% CO_2_ at 37°C for 4 h. The media was discarded after the incubation period, and 200 µL of DMSO was added to each well. We measured the OD at 560 nm.

### 2.7 Statistical analysis

Using Minitab^®^ version 18 (2017), the analysis of variance (ANOVA) test was utilized to examine the results presented as mean ± standard deviation. Post-hoc pairwise comparisons were performed using the Tukey test as in our previous study [21]. For all tests, statistical significance was present in *p*  <0.05.

## 3 Results and discussion

### 3.1 Chemical composition of LEO

**Figure 1:**
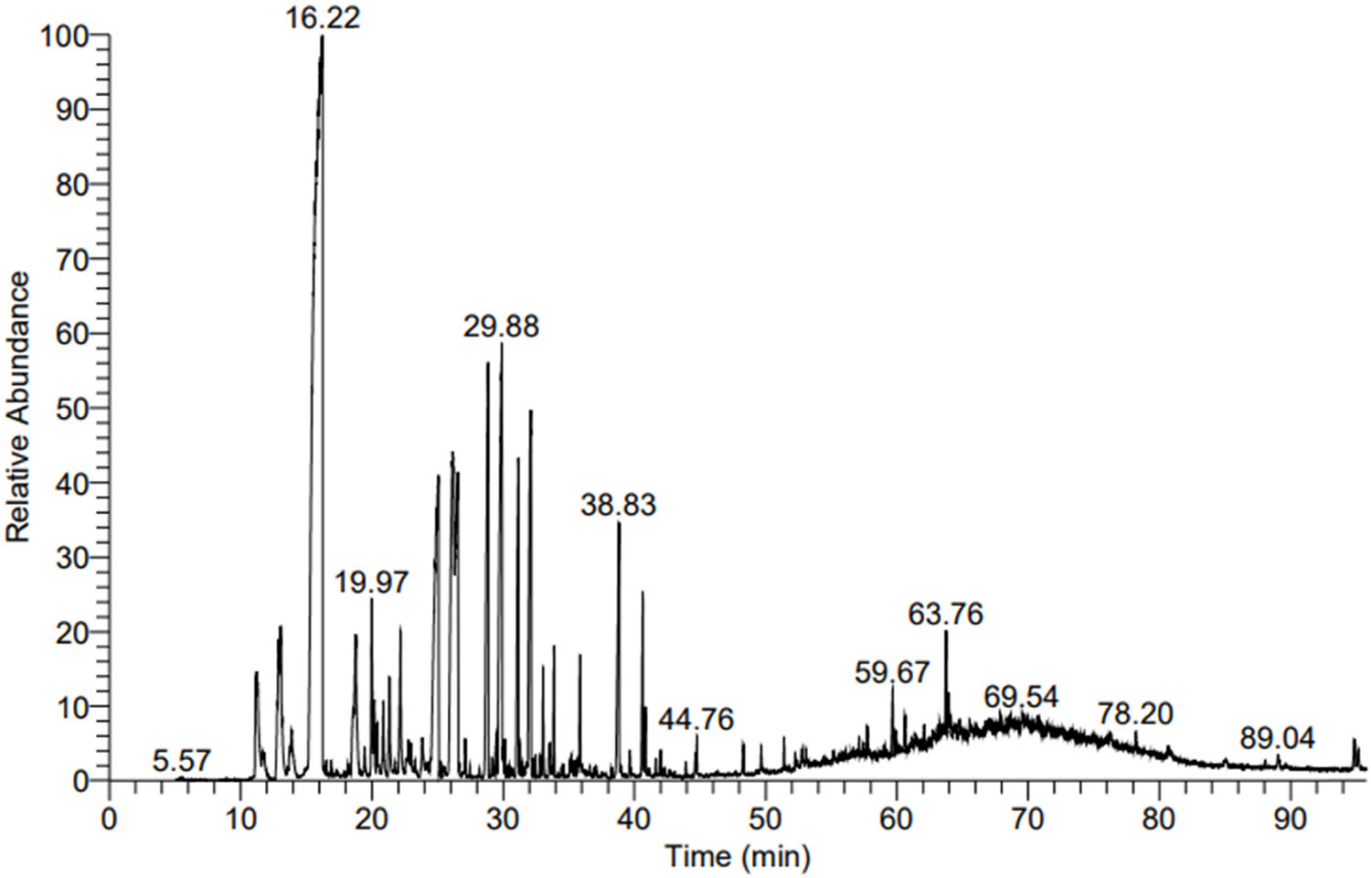
A typical chromatogram of the GC–MS analysis for LEO.

Steam distillation of lemongrass produced a translucent and colorless liquid oil with a lemon-like odor. Figure 1 shows the identified components of the LEO. The volatile contents of the LEO compounds were analyzed by GC–MS, and 30 compounds accounted for more than 0.49% for a total of 90.32%. GC–MS revealed that D-limonene is the dominant compound among others with 25.13%. Many compounds such as neral; linalyl acetate; neral diethyl acetal; limonene oxide, cis-; 2,6-octadienal, 3,7-dimethyl-, (E)-; 5-octyldihydro-2(3*H*)-furanone; (2E)-1,1-diethoxy-3,7-dimethyl-2,6-octadiene were also observed.

From the findings of Hirai et al. [22], Aguilar-Hernández et al. [23], Perdones et al. [6], and Campolo et al. [24], LEO’s most prevalent constituent was limonene, although its concentration was varied. The LEO contains monoterpenes such as D-limonene and limonene oxide, which have been shown to have antiـoxidant properties [28]. For instance, limonene’s antioxidant activity has been found to protect neurons [29], lymphocytes from oxidative stress, and mitochondria from malfunction [25]. This investigation did not discover LEO components such as β ـphellandrene [22], camphene, or sabinene [23], which have been found in previous research. The content of LEO may vary based on the geographic location, environmental variables, developmental stage, plant age, extraction site, harvest time, and extraction technique [26].

### 3.2 DLS and TEM analyses

**Figure 2:**
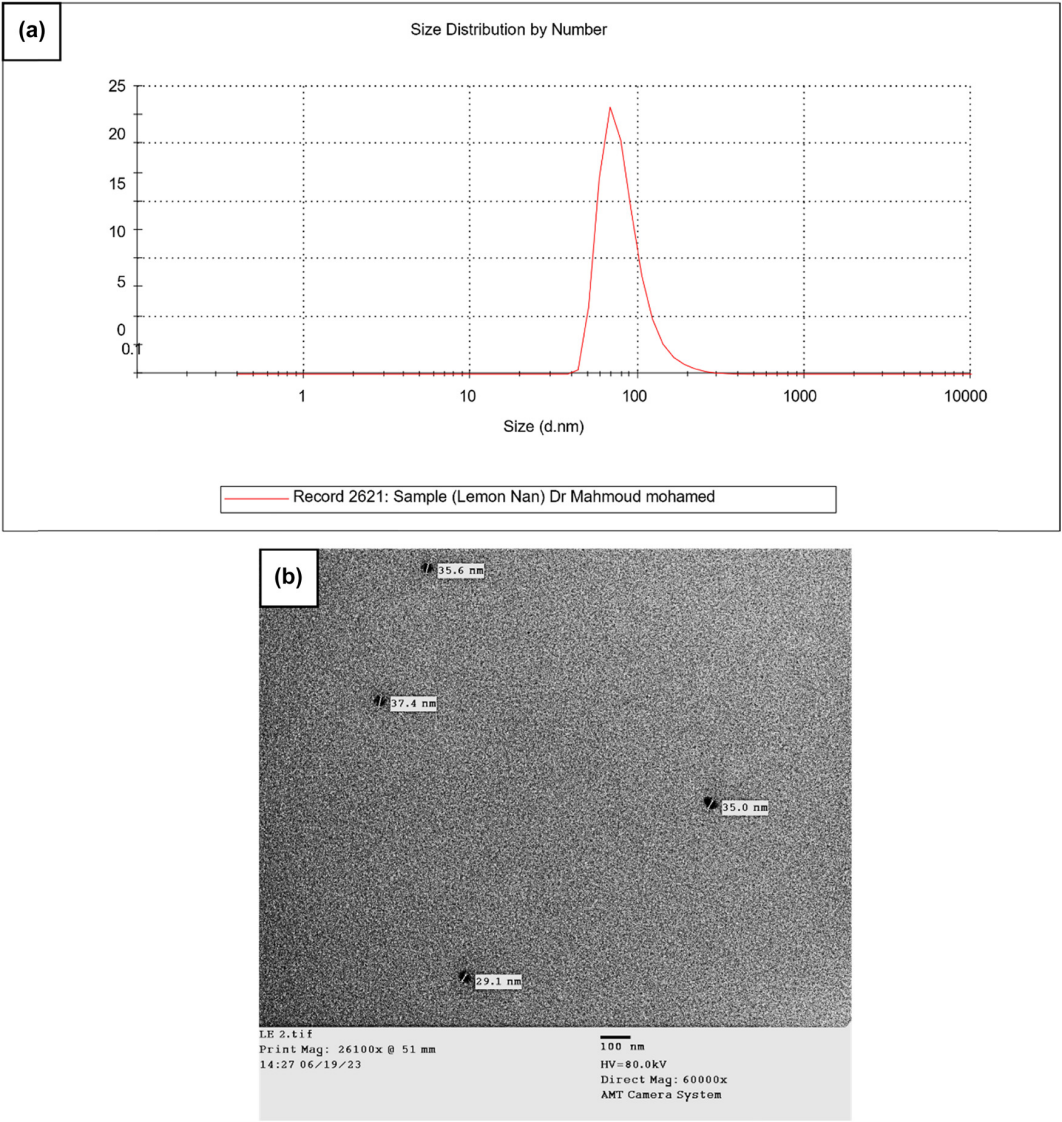
(a) Effect of ultrasonication on the particle size of LEO-NEs prepared by the ultraـsonication method (peak at 91.3 nm; PDI = 0.163). (b) TEM picture of a 30 min ultrasonication-prepared LEO-NEs (size range: 29.1–37.4 nm).

An investigation of droplet size (Figure 2a) and structure at a higher resolution is possible with electron microscopy. The shape of the W/O/W NEs was examined using TEM imaging. Analysis of the TEM images, as shown in Figure 2b, showed a spherical droplet shape with a constant size spanning from 29.1 to 37.4 nm. The TEM measurements of particle sizes were found to be in close agreement with DLS measurements. This signifies that the findings of a low PDI value of 0.163 are corroborated by the TEM pictures, which clearly show the uniform dispersion of W/O droplets inside the exterior water phase. These observations emphasize the W/O/W NE’s potential for a variety of nanotechnology applications by offering insightful information on its stability and structural properties. DLS yielded a particle size of 91 nm, while TEM yielded approximately 30 nm. This difference suggests a convergence of results, as DLS measures the hydrodynamic size of particles in solution, including any solvent shell, and is sensitive to aggregates, while TEM provides a direct image of particles in a dried, solid state, revealing their core size [27].

### 3.3 Antimicrobial activity

**Figure 3:**
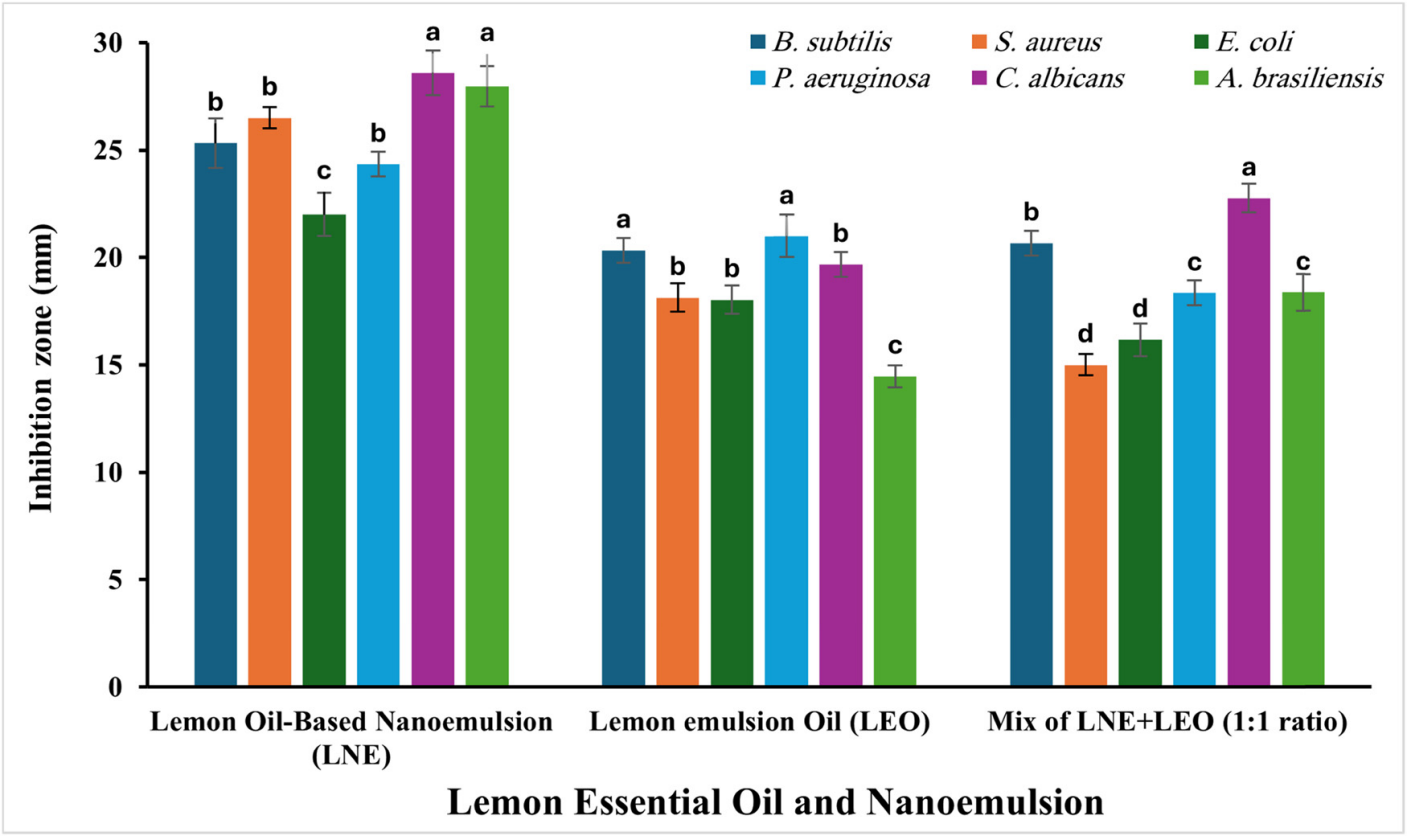
Antimicrobial efficacy of LEO-NEs, LEO, and a mixture of them against different microbial pathogens.

Emulsions and nanoـemulsions containing clove, lemongrass, palmarosa essential oil, or thyme exhibited significant antibacterial efficacy [28,29]. The agar well diffusion method was used to evaluate the antimicrobial activity of LEO-NEs, LEO-E, and their mixture in suppressing the development of numerous pathogens, including *S. aureus*, *B. subtilis*, *P. aeruginosa, E. coli, C. albicans*, and *A. brasiliensis*. The most important action in this probe was verified for LEO-NEs with inhibition zones of 25.33 ± 1.1, 26.5 ± 0.5, 22 ± 1, 24.33 ± 0.5, 28.6 ± 1, and 27.97 ± 0.9 mm for *B. subtilis, S. aureus, E. coli, P. aeruginosa, C. albicans,* and *A. brasiliensis*, respectively (Figure 3). These easily visible inhibition zones’ diameter decreased at LEO-E, where inhibition zones were 20.3 ± 0.57, 18.1 ± 0.66, 18 ± 0.65, 21 ± 1, 19.7 ± 0.57, and 14.5 ± 0.50 mm for *B. subtilis, S. aureus, E. coli, P. aeruginosa, C. albicans,* and *A. brasiliensis*, respectively. On the other hand, inhibition zones of LEO-NEs/LEO mixture of 20.7 ± 1.1, 15 ± 0.5, 16.2 ± 1.1, 18.3 ± 0.57,22.8 ± 1.02, and 18.4 ± 0.95 mm were observed for *B. subtilis, S. aureus, E. coli, P. aeruginosa, C. albicans,* and *A. brasiliensis,* respectively.

Generally, the antimicrobial action of essential oils involves phenolic compounds interacting with porin proteins in the cytoplasmic membrane. This interaction can cause precipitation, leading to ion leakage and other cellular contents, ultimately resulting in cell collapse [30]. Thyme essential oil and other essential oils with thymol and carvacrol terpenes have been demonstrated to have strong antibacterial properties [31]. However, eugenol and citral, the primary constituents of clove, lemongrass, and rosewood essential oils, have been shown to inactivate a wide range of microorganisms [32]. Thus, the final antibacterial activity of a particular essential oil is determined by the concentration and composition of each volatile molecule, which is determined by the kind of plant, growth environment, and essential oil extraction technique. In our investigations, it was observed that NEs considerably influenced their antibacterial action, since NEs demonstrated an elevated and robust inactivation of all pathogenic strains.

It is widely believed that lipophilic antimicrobials in NEs will have enhanced bactericidal effects because they will be able to pass through bacterial membranes more readily. Porins, which are proteins that form channels or pores in the outer membrane of Gram-negative bacteria like *E. coli* and *P. aeruginosa*, allow hydrophilic particles to enter the bacterial cells while acting as a barrier to lipophilic compounds and limiting the entry of compounds based on size [33]. Thus, it is reasonable to assert that the solubilization of lipophilic antimicrobials in NEs will increase the availability of binding sites for interaction with porins. Recent investigations have shown an enhancement in the antibacterial efficacy of limonene-infused NEs, aligning with our observations [34].

Nanoemulsions exhibit antibacterial activity through several mechanisms, including the physical interaction of their sharp edges with bacterial cell walls, which can lead to membrane disruption [35,36]. They also generate reactive oxygen species (ROS), causing oxidative stress and cellular damage even under dark conditions [37–39]. Additionally, nanoemulsions can trap bacteria within aggregated particles, immobilizing them and enhancing the antibacterial effect [40]. Other mechanisms include interference with glycolysis, damage to bacterial DNA, and the release of metal ions that possess intrinsic antibacterial properties [41–43]. Recently, the contribution of nanoemulsions to the generation of nanobubbles has been noted, which can further amplify their antibacterial efficacy [44,45].

### 3.4 Antibiofilm activity

**Figure 4:**
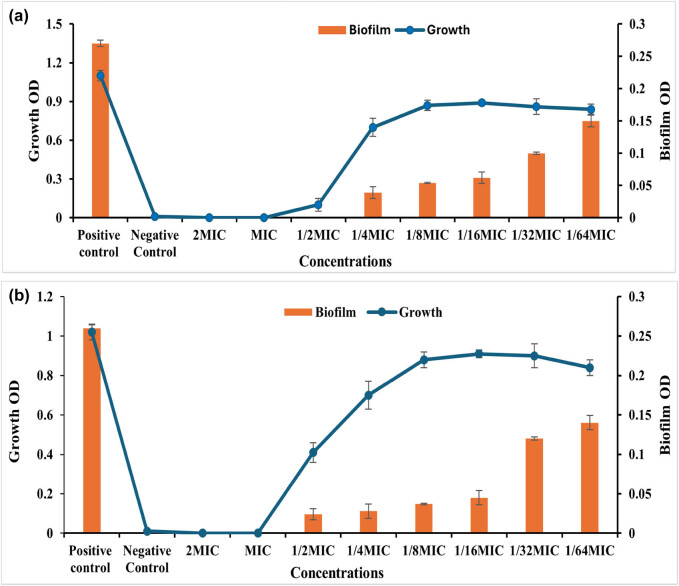
Antibiofilm activity of LEO against the *S. aureus* (a) and *P. aeruginosa* (b) strains.

**Figure 5:**
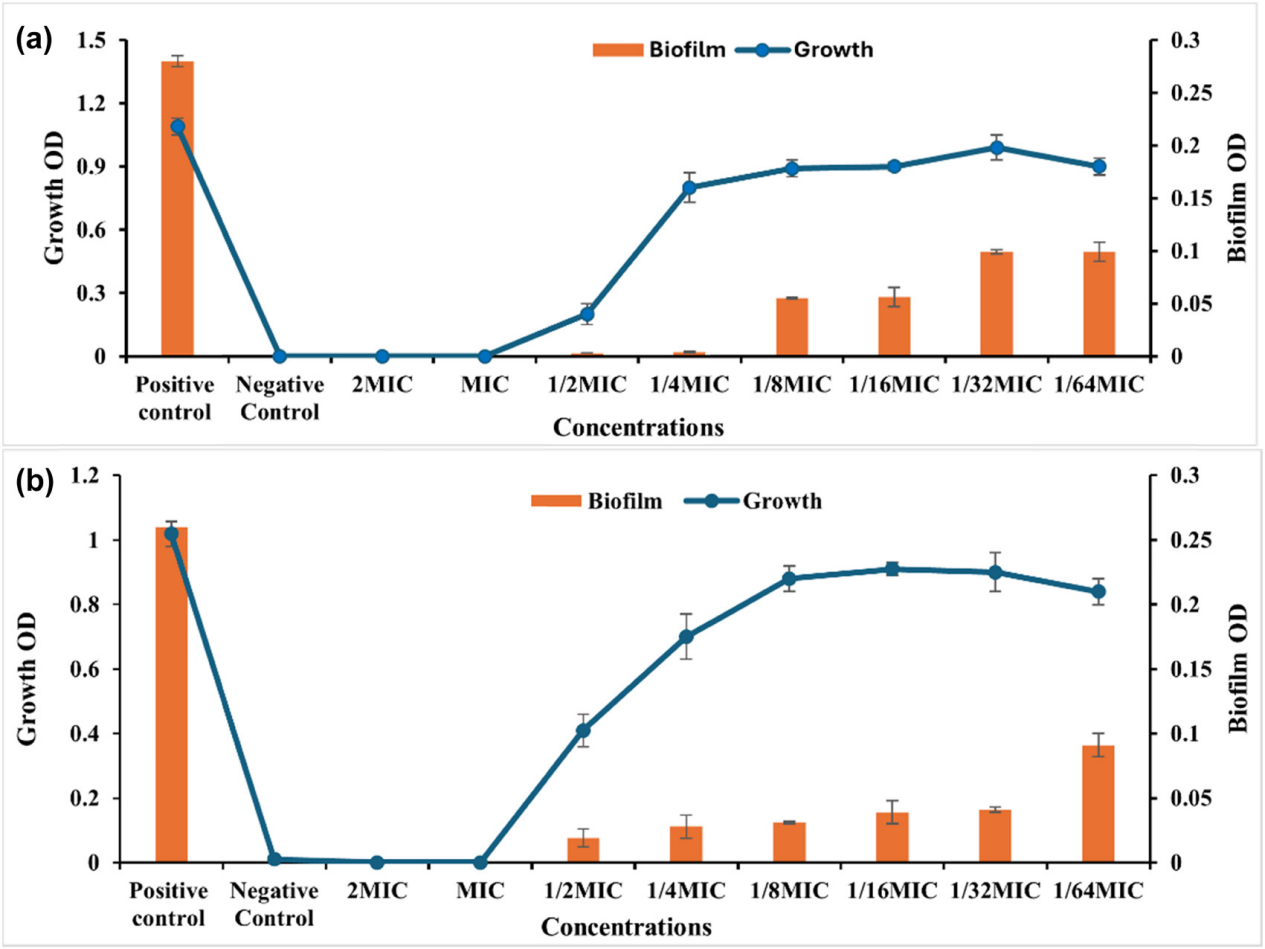
Antibiofilm activity of LEO-NE against the *S. aureus* (a) and *P. aeruginosa* (b) strains.

**Figure 6:**
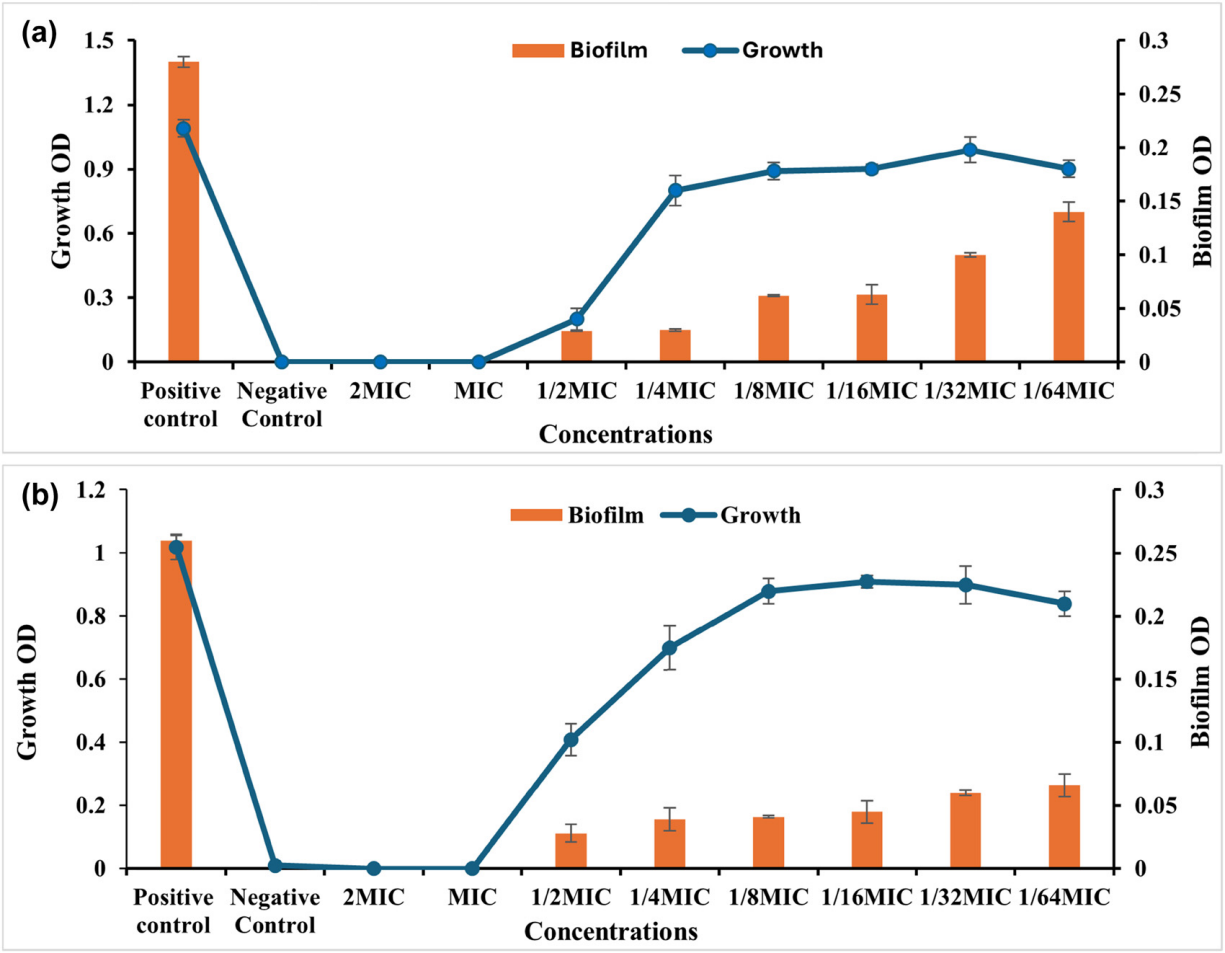
Antibiofilm activity of the mixture of LNE and LEO against the *S. aureus* (a) and *P. aeruginosa* (b) strains.

One of the most effective strategies to counteract the phenomenon of antimicrobial resistance is the use of anti-virulence agents, which work by shifting the focus from microbial survival, which is what drives the development of resistance genes in bacteria to the microbial virulence factors, which are thought of as the agents’ weapons that cause the pathogenicity to the host [46]. A complex polymer matrix known as a biofilm is created by the microbial population as a defense against antimicrobial agents and the host immune system. Microorganisms also use the matrix to exchange nutrients and water. One significant virulence weapon that ants may use as a good target for their virulence agents is biofilms. After assessing the anti-bacterial activity of LEO-NEs, LEO, and their combination versus proliferation of planktonic cells of *S. aureus* and *P. aeruginosa,* this study tested LEO-NEs, LEO, and their combination at sub-inhibitory concentrations to prevent biofilm formation by these strong biofilm-producing strains (Figures 4–6).

The results shown in Figure 4 demonstrated that LEO exhibited antiـbiofilm activity versus both *S. aureus* and *P. aeruginosa*. The tested concentrations of 25.0, 12.50, 6.250, 3.125, and 1.560 µg/mL corresponding to 1/4, 1/8, 1/16, 1/32, and 1/64 of the MIC, respectively, inhibited the biofilm formation of *S. aureus* by 85.55, 80.0, 77.03, 62.96, and 44.44%, respectively, in a dose-dependent way, without influencing the proliferation of planktonic cells (Figure 4a). While the values 50, 25, 12.5, 6.25, 3.125, and 1.56 µg/mL, corresponding to 1/2, 1/4, 1/8, 1/16, and 1/32 of MIC, respectively, inhibited biofilm formation of *P. aeruginosa* by 90.76, 89.23, 85.76, 82.69, 53.84, and 46.15%, respectively (Figure 4b).

A similar pattern is observed in the results presented graphically in Figure 5, which showed strong antibiofilm activity of LEO-NEs against both biofilm-forming *S. aureus* and *P. aeruginosa* pathogens, where the concentrations 12.5, 6.25, 3.125, 1.56, 0.78, and 0.39 µg/mL representing 1/2 ,1/4, 1/8, 1/16, 1/32, and 1/64 of MIC suppress the biofilm formation of *S. aureus* by 98.92, 98.57, 80.35, 80.0, 64.64, and 64.64%, respectively (Figure 5a). Also, 6.25, 3.125, 1.56, 0.78, 0.39, and 0.195 µg/mL representing 1/2, 1/4, 1/8, 1/16, and 1/32 of MIC inhibited biofilm formation of *P. aeruginosa* by 92.62, 89.23, 88.07, 85, 84.23, and 65%, respectively (Figure 5b).

On the other hand, the results represented graphically in Figure 6 showed antibiofilm activity of LEO-NEs and LEO combination’s against biofilm-forming *S. aureus* and *P. aeruginosa* cells where the concentrations 25, 12.5, 6.25, 3.125, 1.56, and 0.78 µg/mL corresponding to 1/2, 1/4, 1/8, 1/16, 1/32, and 1/64 of MIC inhibited the biofilm production of *S. aureus* by 89.64, 89.28, 77.85, 77.5, 64.28, and 50%, respectively in a dose-dependent way, with no effect on the proliferation of planktonic cells (Figure 6a). Also, 25, 12.5, 6.25, 3.125, 1.56, 0.78 µg/mL representing the 1/2, 1/4, 1/8, 1/16, and 1/32 of MIC inhibited biofilm formation of *P. aeruginosa* by 89.23, 85, 84.23, 82.69, 76.92, and 47.61%, respectively in a dose-dependent way without influencing the proliferation of planktonic cells (Figure 6b).

Generally, our findings demonstrated that the formation of bacterial biofilm was inhibited by LEO-NEs, LEO, and a combination of them; however, the inhibitory impact of the essential oil emulsified as LEO-NE was significantly greater than that of the essential oil and the combination of them.

The first crucial step in preventing the formation of biofilms is to impede adhesion [47]. The essential oil NE’s anti-biofilm properties can be explained by its capacity to inhibit the attachment of bacteria to surfaces. Inhibiting the production of biofilm was the first step toward reducing the harmful impact of bacteria [27]. The formation of biofilm was significantly inhibited by the treatment of essential oils, particularly the nanoـemulsified essential oil. This is why their application in the culinary industry is eagerly anticipated [48]. The biofilm is crucial for pathogens in mitigating the efficacy of antibacterials; hence, food-based nanoـemulsions of essential oils may serve as an effective alternative for controlling foodborne infections.

### 3.5 Antioxidant activity

**Figure 7:**
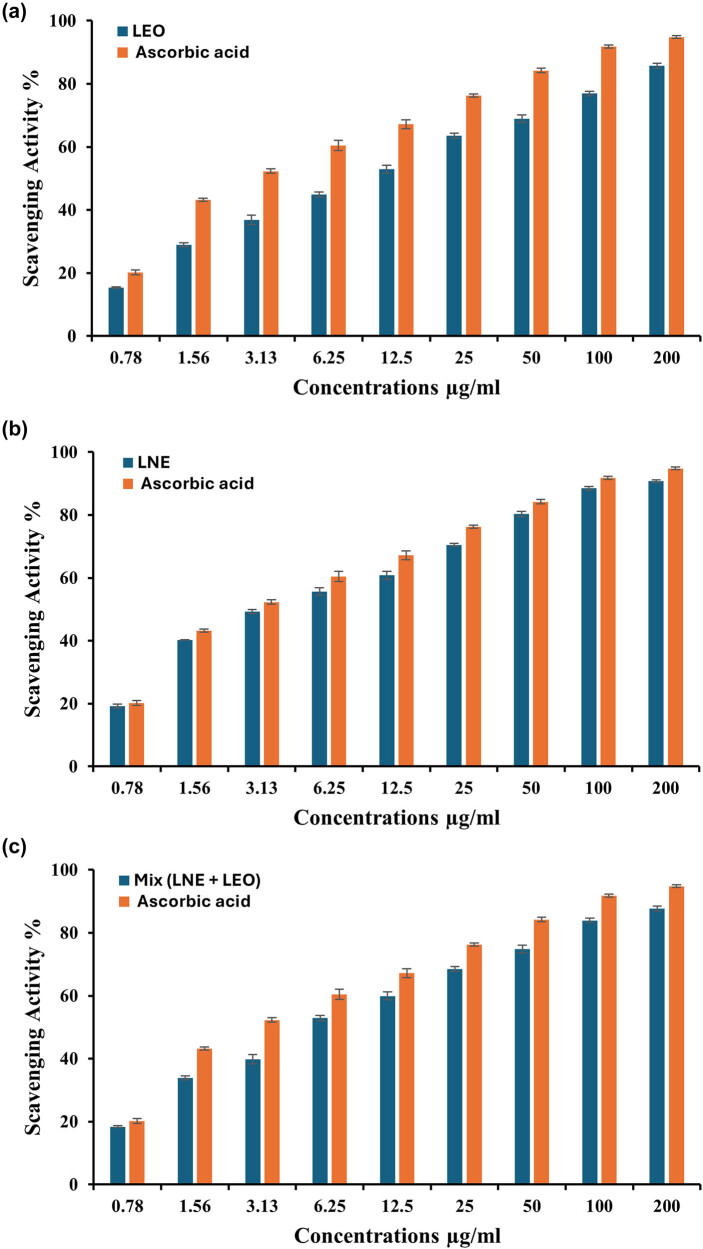
Antioxidant activity of LEO (a), LEO-NEs (b), and a mixture of them (c) compared to ascorbic acid (control).

Finding new compounds with antioxidant properties is crucial for the medical and health industries. Among the various diseases that free radicals can cause are neurological disorders, cancer, and heart diseases [49]. Therefore, the discovery of novel antioxidant molecules is essential to the creation of fresh approaches to treating and preventing chronic illnesses. Nanoparticles are considered promising antioxidants because of their distinctive physicochemical properties, including their small size and high surface-to-volume ratio. These characteristics enhance their activity and facilitate their interaction with biological systems to neutralize free radicals [50]. The effectiveness of LEO-NEs, LEO, and a combination of them in scavenging free radicals was assessed in this investigation using the DPPH assay (Figure 7). The antioxidant capacity of LEO-NEs, LEO, and their combination was dependent on concentration; the higher concentrations resulted in increased activity, and lower concentrations led to decreased activity [51].

The DPPH assay method revealed that 200 µg mL^−1^ LEO had the maximum scavenging action, with a scavenging percentage of 85.7 ± 0.8%. This difference is not statistically significant (*p* ≥ 0.001) when compared to ascorbic acid (94.8 ± 0.4%), which was used as a positive control (Figure 7a). Furthermore, at a dosage of 100 µg mL^−1^, there was no significant difference in the antioxidant activity between LEO and ascorbic acid, with scavenging percentages of 76.9 ± 0.7% and 91.8 ± 0.5%, respectively. The concentration of 0.78 µg mL^−1^ with a scavenging percentage of 15.39 ± 0.25% and the concentration of 1.56 µg mL^−1^ with a scavenging percentage of 28.9 ± 0.6% showed the lowest scavenging activity when compared to ascorbic acid at the same concentrations (20.2 ± 0.8% and 43.2 ± 0.5%, respectively) (Figure 7a).

A similar pattern was seen when using the DPPH assay, which showed that 200 µg mL^−1^ LEO-NEs had the highest percentage of scavenging action (90.77 ± 0.4%). Statistical analysis reveals that the difference between this and the positive control, ascorbic acid (94.8 ± 0.4%), is not significant (*p* ≥ 0.001) (Figure 7b). Moreover, at 100 µg mL^−1^ dosage, LEO-NEs and ascorbic acid did not significantly differ in their antioxidant activity, with scavenging percentages of 88.5 ± 0.6 and 91.8 ± 0.5%, respectively. When compared at the same concentrations, the ascorbic acid effects (20.2 ± 0.8 and 43.2 ± 0.5%, respectively), and the concentration of 0.78 µg mL^−1^ with a scavenging percentage of 19.2 ± 0.6% and the concentration of 1.56 µg mL^−1^ with a scavenging percentage of 40.2 ± 0.1% displayed the lowest scavenging activity (Figure 7b).

However, 200 µg mL^−1^ mixture of LEO and LEO-NEs showed the highest scavenging effect, with a scavenging percentage of 87.7 ± 0.8%, according to the DPPH assay. Based on statistical analysis, there is no significant difference (*p* ≥ 0.001) between this and the positive control ascorbic acid (94.8 ± 0.4%) (Figure 7c). Furthermore, the mixture of LEO and LEO-NEs compared with ascorbic acid showed similar antioxidant efficacy at a 100 µg mL^−1^ dosage, with scavenging percentages of 83.9 ± 0.7 and 91.8 ± 0.5%, respectively. In contrast to ascorbic acid at identical doses (20.2 ± 0.8 and 43.2 ± 0.5%, respectively), the least amount of scavenging activity was seen at 0.78 µg mL^−1^ with a scavenging percentage of 18.39 ± 0.25% and at 1.56 µg mL^−1^ with a scavenging percentage of 33.9 ± 0.6% (Figure 7c).

Antioxidants can exhibit pro-oxidant behavior under specific conditions, such as high concentrations or in the presence of transition metals. In the case of LEO-NEs, the nanoemulsion may act as an antioxidant at low concentrations by scavenging ROS in normal cells, while at higher concentrations or in specific cellular environments (e.g., cancer cells or bacteria), it can induce ROS generation and oxidative stress. This dual behavior was reported by Kurutas [52], which demonstrated that plant-based nanoemulsions can switch from antioxidant to pro-oxidant activity depending on the concentration and cellular context. Moreover, LEO-NEs may selectively induce ROS generation in cancer cells or bacteria due to their altered redox homeostasis compared to normal cells. Cancer cells, for instance, often exhibit elevated baseline ROS levels, making them more susceptible to further ROS-induced damage. This selective cytotoxicity was supported by Abd-Rabou and Edris [53], who showed that the frankincense essential oil nanoemulsion can exploit the redox imbalance in cancer cells to induce apoptosis through ROS-mediated pathways. The nanoemulsion formulation itself can enhance the pro-oxidant effects of LEO-NEs, by improving the delivery and intracellular uptake of bioactive compounds. This can lead to localized ROS generation and oxidative stress in target cells [54].

### 3.6 Cytotoxicity

Using MTT dye in a colorimetric experiment, the cytotoxic potential of NEs loaded with bioactive compounds was evaluated. The results showed that the cell viability of cell lines treated with NEs of bioactive substances was similar to that of the negative control, indicating the non-toxicity and safety of NEs for various applications. Several normal and cancer cell lines were employed for the material’s *in vitro* investigations (Table 1). The efficiency with which the purple MTT tetrazolium dye was converted into its insoluble formazan metabolite by NADPH-dependent oxidoreductase enzymes in cells was assessed [55]. Cell viability should rise with growth, fall with anticancer treatments, and stay constant (or plateau) with cytostatic treatments. Since the control sample produces healthy cells with 100% vitality, it can be used to estimate the cell viability percentage.

**Table 1:** Cytotoxicity and anticancer activity of LEO-NEs and LEO-E against WI-38, PC3, and Hep-G2

Sample	Concentration (µg/mL)	Toxicity (%)		
		WI-38	PC3	HepG-2
LEO	**1,000**	97.60	97.24	97.29
	**500**	89.12	95.80	97.19
	**250**	61.27	93.54	97.14
	**125**	28.87	88.17	75.19
	**62.5**	0.00	59.39	38.60
	**31.25**	0.00	7.81	4.86
	**IC**_**50**_ **± SD**	207.2 ± 2.87	70.94 ± 1.06	86.87 ± 1.27
LEO-NEs	**1,000**	97.44	97.38	97.39
	**500**	91.15	95.71	97.29
	**250**	73.57	75.07	97.24
	**125**	21.36	40.65	64.56
	**62.5**	0.08	9.53	17.99
	**31.25**	0.16	0.36	0.15
	**IC**_**50**_ **± SD**	192.07 ± 2.99	170.09 ± 7.35	105.06 ± 1.15

To check the biosafety of both LEO-NEs and LEO-E, the cytotoxicity of it against the normal cell line is required. The cytotoxicity of LEO-NEs and LEO-E was carried out on the WI-38 normal cell line, as shown in Table 1. The results illustrated that IC_50_ of LEO-NEs and LEO-E toward the WI-38 cell line was 192.07 ± 2.99 and 207.2 ± 2.87, respectively. Typically, if the IC_50_ is equal to or more than 90 μg/mL, the substance is categorized as non-cytotoxic [56]. Consequently, both LEO-Nes and LEO-E are deemed to be safe for use.

Additionally, anticancer activity was assessed for LEO-NEs and LEO-E against the human prostate cancer cell line (PC3) and human liver cancer cell line (HepG-2), as illustrated in Table 1. Table 1 shows the percentages of inhibition of LEO-NEs and LEO-E against PC3 and Hep-G2 cancerous cell lines at different concentrations. Inhibition percentages at increasing concentrations were shown by the data. Consequently, at 125 µg/mL, LEO-NEs showed very strong cytotoxic efficacy against PC3 and HepG-2 cell lines, with percentage inhibitions of 21.36 and 40.65%, respectively. The curves created by charting the percentages of cell growth against the LEO-NE concentration (µg/mL) provided the results. As a result, as the extract concentration and IC_50_ values drop, the effect of cytotoxic activity increases. The results revealed that the IC_50_ values of LEO-NEs toward PC3 and HepG-2 cell lines were 170.09 ± 7.35 and 105.06 ± 1.15 µg/mL, respectively. To compare the outcomes of the studied samples, the LEO was used with IC_50_ values of 70.94 ± 1.06 and 86.87 ± 1.27 µg/mL for PC3 and HepG-2 cell lines, respectively (Table 1).

Another study assessed the cytotoxic effects of clove oil NEs on BEAS-2B and L02 cell lines. After 24 h of treatment with 0.025 mg/L concentration, survival rates exceeded 90% [57]. According to Kuar et al. [58], that emulsions were found to show no cytotoxicity against cells, with cell viability reported to be >85% in all tested concentrations (3.125 to 50 μg/mL) and replications. The mechanisms proposed for cytotoxic activities are ROS generation [59], oxidative stress [60], wrapping the cells by aggregated nanomaterials [61], disruption of membrane integrity [62], DNA fragmentation [63], alteration of protein structures, and chromosomal aberration [64].

## 4 Conclusions

In conclusion, this study successfully developed LEO-NE as a promising alternative to traditional citrus essential oils, addressing their limitations in biological stability and water solubility. GC–MS analysis confirmed D-limonene as the primary compound in LEO, which contributes to its bioactive properties. Characterization via TEM and DLS revealed that LEO-NE consists of uniform spherical droplets, enhancing its application potential. Notably, LEO-NE demonstrated significant antimicrobial activity against various pathogens, including *B. subtilis*, *S. aureus*, and *E. coli*, with impressive inhibition zones. Its strong antiـbiofilm activity, particularly versus *S. aureus* and *P. aeruginosa*, highlights its potential in combating biofilm-related infections. Furthermore, LEO-NE exhibited potent antioxidant activity and displayed significant anticancer effects against PC3 and Hep-G2 cell lines. Overall, the findings indicate that LEO-NE has substantial potential for applications in medical and pharmaceutical fields, paving the way for further research and development.
